# SARS-CoV-2 infection dynamics in the tourism season 2020 in North Frisia, Germany

**DOI:** 10.3389/fepid.2022.1029807

**Published:** 2022-12-01

**Authors:** Timo Greiner, Annette Aigner, Peter Tinnemann

**Affiliations:** ^1^Berlin School of Public Health, Charité – Universitätsmedizin Berlin, Berlin, Germany; ^2^Institute of Biometry and Clinical Epidemiology, Charité – Universitätsmedizin Berlin, Berlin, Germany; ^3^Health Protection Authority of the City of Frankfurt am Main, Frankfurt am Main, Germany; ^4^Institute for Social Medicine, Charité – Universitätsmedizin Berlin, Berlin, Germany

**Keywords:** SARS-CoV-2, COVID-19, tourism, transmission, occupation

## Abstract

**Background:**

International tourist activities including air travel, holiday on cruise ships, and Après-ski parties played a prominent role in the early spread of the SARS-CoV-2 pandemic. However, the effects of national tourism on infection dynamics are unclear.

**Methods:**

Data were analyzed from the health authorities in North Frisia, the northernmost district in Germany with prominent tourist hotspots such as *Sylt, Amrum*, and *Föhr*. Data were available for the time period April 2020–November 2020.

**Results:**

During the tourist season (May–October 2020), PCR-confirmed SARS-CoV-2 case numbers were low with 0 to 10 cases per day. Case numbers rose in September and peaked end of October (2nd wave). Among the confirmed cases, 13 persons were returning travelers and none were national tourists. Overall, only a small proportion of cases were related to individuals with presumed tourist contact.

**Conclusion:**

In summer 2020, the arrival of a large number of tourists apparently did not increase local case numbers, and tourism-related outbreaks were not reported. Thus, tourism presumably did not contribute substantially to SARS-CoV-2 infection dynamics in North Frisia. However, incidences were low countrywide and protective measures were in place.

## Introduction

In December 2019 the novel coronavirus Severe Acute Respiratory Syndrome Corona Virus-2 (SARS-CoV-2) emerged in Wuhan, China, and since then spread worldwide (1). The World Health Organization (WHO) declared COVID-19 as a public health emergency of international concern status on January 30th and declared the status of a pandemic on March 11th (2).

In Germany, the course of the pandemic was defined by phases. In *Phase 0* (January and February 2020) sporadically occurring cases in regional outbreaks were reported (*n* = 167). It was followed by *Phase I*, known also as the “first wave” (March to May 2020). Overall, above 175,000 cases were reported and mostly nursing homes and hospitals were affected. In the longer *Phase 2* during summer (May to September 2020) around 112,000 cases were reported, among these mostly travel-associated cases and cases in factories. In *Phase 2*, cases with severe symptoms occurred less frequently compared to *Phase 1*. The “second wave” occurred in *Phase 3* (September 2020 to February 2021), where more than two million cases were reported. Persons of all age groups were affected and cases with severe symptoms occurred more frequently compared to *Phase 2* (3, 4).

In 2020, the main non-pharmaceutical interventions (NPI) for SARS-CoV-2 infection were masks (e.g., surgical or FFP2 masks), social distancing (distance between individuals of at least 1.5 m), handwashing, contact tracing, and testing with PCR tests (5). Other protective measures were temporal shutdowns (e.g., hotels, restaurants, bars), recommendation to work in home office where possible, and travel restrictions (6).

Non-pharmaceutical interventions were put in place on March 16th 2020 (“first lockdown”) and included e.g., closure of schools and kindergartens, borders were closed on March 17th 2020, and tourism was banned from March 19th 2020. Returning travelers were sent into quarantine from April 10th 2020 and masks were made mandatory from April 29th 2020 (2). In March and April 2020 hotel visits were only allowed for business travel, and visiting a secondary home in North Frisia was forbidden (7). The first lockdown ended at the beginning of May 2020. Gastronomy opened again on May 11th and new tourism-specific countermeasures included hygiene concepts (e.g., regular cleaning of surfaces, avoiding of crowd formation) and registering contact data from guests. As well, in gastronomy distance between tables had to be increased to 2 m (8).

In November 2020 a so-called “light” lockdown was put in place due to rising case numbers. This lockdown included closures of hotels and gastronomy, but schools and most commerce remained open. The official second full lockdown with closures of schools and commerce followed in December 2020 (9).

International travel facilitated the spread of the virus and tourism has been linked to the first cases in Europe. A prime example for tourism's potential contribution to the transmission of SARS-CoV-2 is the Austrian ski town Ischgl, where Après-Ski gatherings in bars turned into so-called “super-spreader events.” These lead to the spread of the virus to Germany, Iceland, the United States of America, and other countries (10). Other examples of outbreak events were reported on cruise ships and flights with tourist groups (11–13). Apart from these outbreak events related to tourism, travel activity in general was found to be associated with a higher incidence of SARS-CoV-2 cases. For example, one study based on data from more than 90 countries found a positive correlation between international tourism and cumulated levels of SARS-CoV-2 cases (14). It is important to note that restrictions put in place worldwide, including travel restrictions, had severe economic consequences for the international and national tourism sector (15).

North Frisia is the northernmost administrative district of Germany and belongs to the federal state Schleswig-Holstein. North Frisia has around 500 km coastline at the North Sea and prominent tourist regions. The district has a low population density of 80 persons per km^2^ and a total of 167,147 inhabitants living in 133 parishes as at 31st December 2020. The parishes range in size from 11 to 23,189 inhabitants (median: 570). Around 82% of the population live on the mainland. Five coastal islands in the western part of North Frisia (*Sylt, Föhr, Amrum, Pellworm*, and the peninsula Nordstrand), with *Sylt* being the largest island, are highly frequented tourist destinations. There are 1 to 251 hotels (median: 3.5) in 86 parishes (64.7%), and the parishes with the highest number of hotels are *Sylt* (251), *Sankt Peter-Ording* (153), and *Wyk* auf *Föhr* (85). In 2020, 1,163 out of 1,211 hotels were open for tourists between May and November (96%) (16).

So far, the contribution of national tourism in Germany, and particular this coastal region, on virus spread and beyond district borders is unknown. In this study, we analyzed data from the local public health authorities of North Frisia for the time period from April 2020 to November 2020. Visiting the region for tourism was only allowed between May and October, while in April and November hotels were shut down by governmental order. We assessed confirmed and suspected cases in the district, in order to analyze a possible impact of touristic activity on the SARS-CoV-2 infection dynamics in North Frisia.

## Materials and methods

### Data and data sources

Data on SARS-CoV-2 PCR positive cases and their related contact persons are collected by the public health authorities in North Frisia routinely according to the infection protection act (Infektionsschutzgesetz, IfSG). Data were available from April 2020 to November 2020. The dataset contained information on all individuals were isolation was ordered due to a positive PCR-test for SARS-CoV-2 infection (“positive case”) or quarantine was ordered due to a close contact with a person that was tested positive (“contact person”; Note: other types of test results such as from rapid antigen-tests were not reported). Therefore, both, *PCR-confirmed cases* and all *quarantined persons*, were considered in this study. The data were provided in a pseudonymized form and contained the following items: age, test date and result, begin and end of quarantine or isolation, occupation, place of work, responsible public health authority (if not the public health authority in North Frisia), outbreak setting, previous traveling, and detection of virus mutants. In addition, free text with information, e.g., on where transmission took place, was available in some of the data sets.

Outbreaks in this study were defined as groups of ≥2 cases that were epidemiologically connected.

Data on tourism activity in North Frisia were derived from the website of the statistical office (16). These data contain the number of arriving tourists, number of overnight stays, and the origin country of tourists who arrived in hotels with ≥10 beds and without camping – per month. The average duration of stay (in days) was given as overnight stays divided by the number of arriving tourists.

### Approaches for the reconstruction of virus transmission

#### Place of transmission

Information on where persons presumably had contact to an infected person (e.g., in the same household or at work) was reported originally in the database. However, this was not routinely done and information was only available in <10% of persons.

The reported categories were as follows: same household, medical settings, work, school/kindergarten, gastronomy, private meetings and gatherings, nursing home, driving together (car, bus, plane, etc.), other settings (e.g., shopping or participating at a seminar), hotel.

#### Possible settings of infection

As the information on the presumed place of transmission was sparse, we attempted to create a variable called “setting” that was supposed to reflect where persons spend a substantial amount of time during the day and where they might have increased contacts to infected persons. The *setting* of infection was derived based on information on occupation and workplace with the following categories:

1) *Tourism and gastronomy*—with the categories *hotel* (jobs in hotels, e.g., reception or cleaning personnel), *other tourism* (e.g., jobs in tourist information centers), and *gastronomy* (e.g., jobs in restaurants, bars, cafés, etc.).
2) *Medicine*—including persons working or staying in hospitals, rehabilitation centers, physician practices with the categories *staff* (e.g., physicians, nurses, dentists, assistants, cleaners, janitors, drivers), *patients* and *visitors*.
3) *Nursing homes*—including nursing homes for elderly, children, handicapped and workshops for handicapped. Here persons were divided into *staff* and *residents*.
4) *Education*—including *staff* (e.g., teachers, janitors, secretaries, etc.) and *children/students* in kindergartens, day-care centers, schools, professional schools and universities.
5) *No work*—including retired persons, unemployed persons, infants, persons on maternity leave or sick leave or holiday.
6) *Other work settings*. This includes various types of occupation such as technicians, construction workers, food processing workers and others.

#### Occupation

Additionally, the information on occupation only was considered and coded according to the *International Standard Classification of Occupations 2008* (ISCO-08) (17) in order to study which occupational groups were at risk of SARS-CoV-2 infection. For example, a nurse would be coded as 2221 (major group 2: professionals, sub-major group 22: health professionals, minor group 222: nursing and midwifery Professionals, unit group 2221: nursing professionals).

### Statistical analysis

Line lists were created from the case table. Incidences were plotted as epidemic curves and as 7-day moving average. All continuous variables are presented / displayed as arithmetic means ± standard deviation or median [range], categorical variables as absolute and relative frequencies.

Although some data entries were based on the same individual, each entry was considered to be independent, as the order for a quarantine is also independent from another.

Excel was used for data handling and cleaning. Data analysis was performed with R version 4.0.5 (18) with the following packages: tidyverse (19), Incidence2 (20), arsenal (21).

## Results

### SARS CoV-2 infections in North Frisia

**Figure 1:**
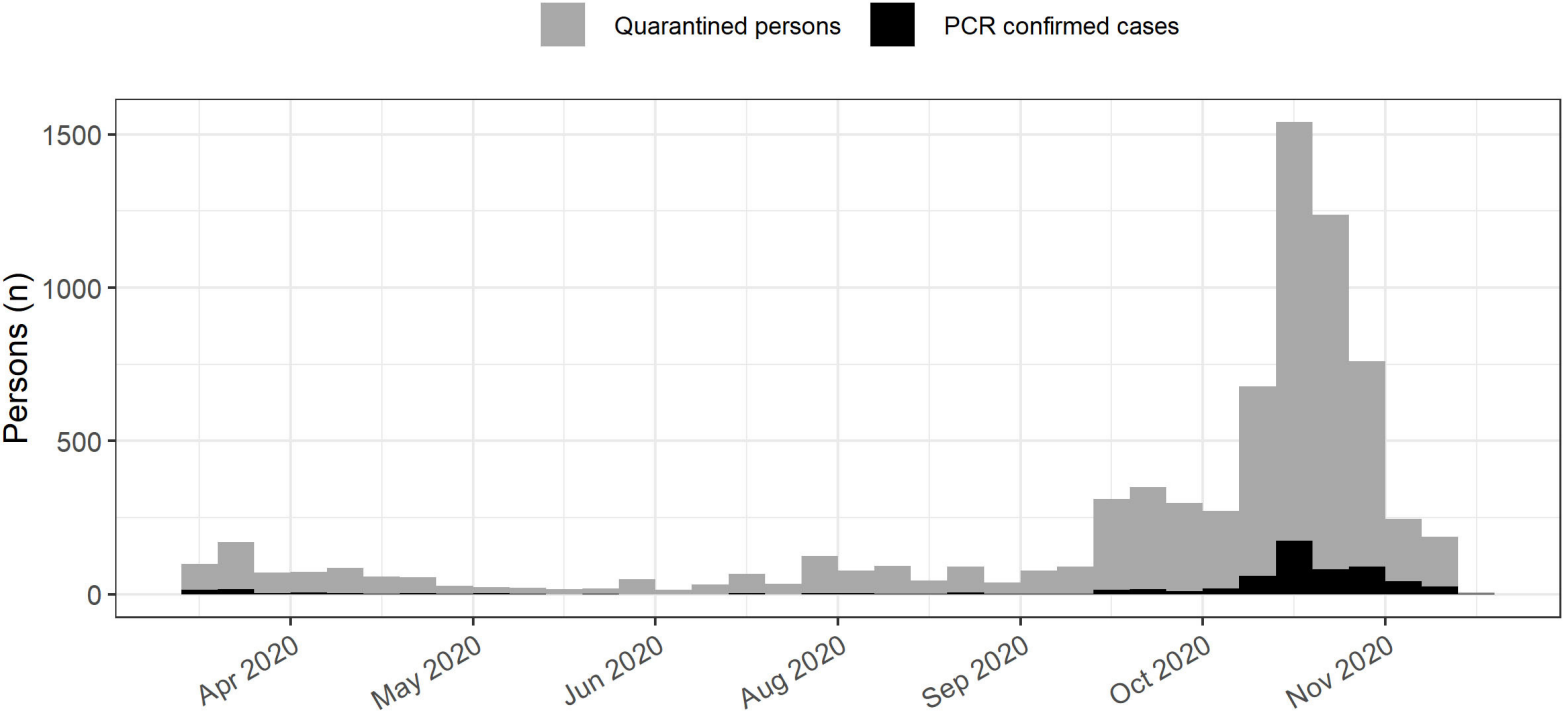
SARS-CoV-2 infections in North Frisia. SARS-CoV-2 cases (black) and quarantined persons (gray) in North Frisia from April 2020 to November 2020.

**Figure 2:**
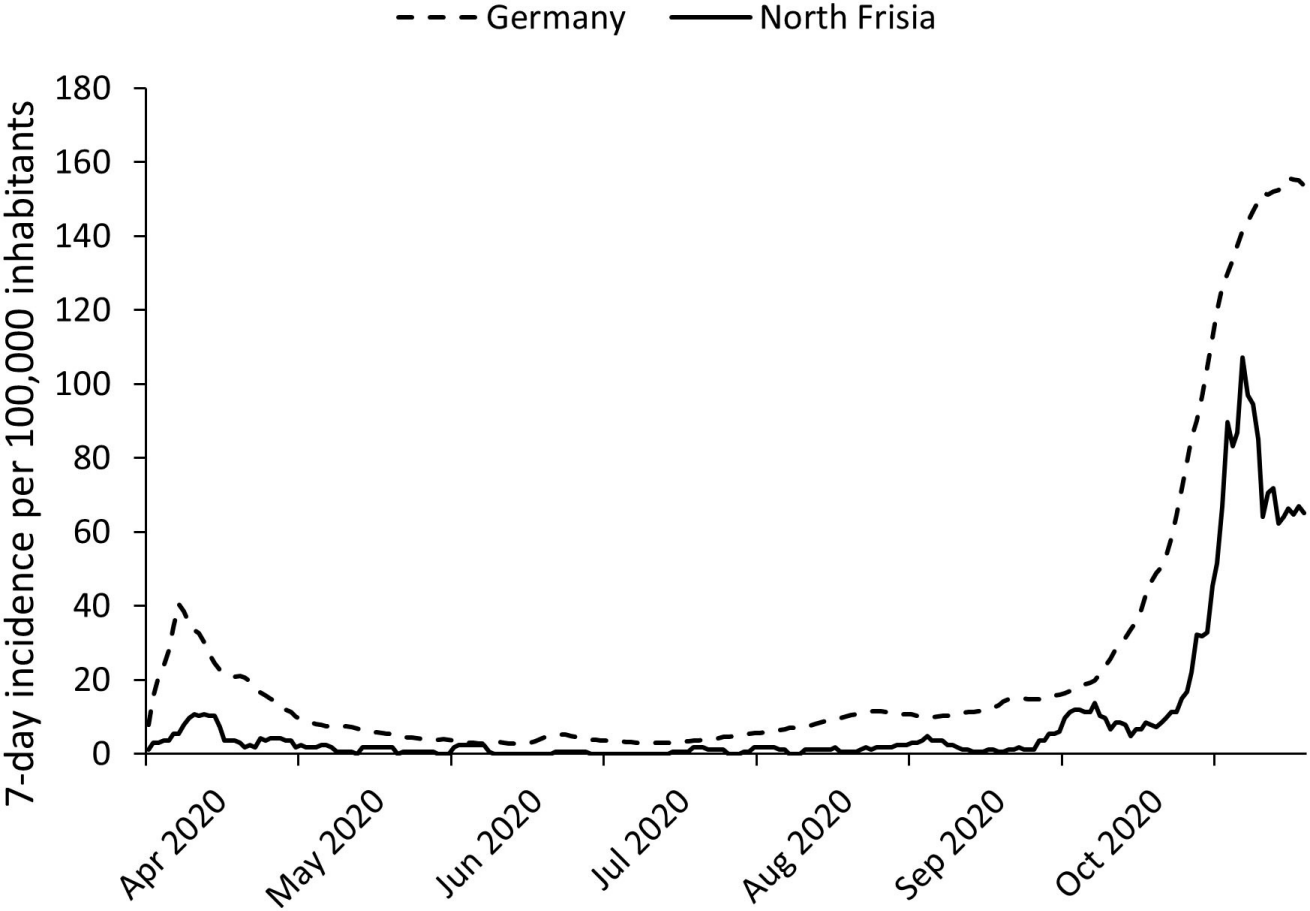
SARS-CoV-2 incidences in Germany and in North Frisia. Seven-day incidences for North Frisia (solid line) and Germany (dotted line) between April 2020 and November 2020.

The dataset contained 7,440 observations from 7,296 persons, as 138 persons were quarantined twice, and three persons three times. The first PCR-confirmed cases of SARS-CoV-2 infections in North Frisia were detected in March 2020. Between 0 and 10 cases per day were reported until mid-October. Therefore, throughout the summer, incidences were low in both, North Frisia and Germany as a whole, but the 7-day incidences were mostly lower in North Frisia. Case numbers only began to rise markedly in late October, which was also the end of the tourist season. A “light” shutdown was put into place on November 2nd. In North Frisia incidences were around 110 cases per 100,000 inhabitants in early November (Figures 1, 2).

In the study population there were slightly more women than men among the quarantined persons (50.7%), with an average age of 32.6 years (SD: 20.6 years), which ranged from infants (0 years) to high age (100 years). The majority of quarantined persons were reported for the mainland (85.2%), and for the vast majority of individuals quarantine was ordered (89.2%). Overall, 611 cases were confirmed by PCR tests (8.2%). Among these, men were slightly predominant (52.3%), with an average age of 38.2 years (SD: 21.3 years), again ranging from infants (1 years) to high age (93 years; Table 1). During the whole time period 3 persons died due to or with a SARS-CoV-2 infection, resulting in a case fatality rate (CFR) of 0.5%. The mean age of the deceased was 86.0 years (SD: 1.0 years). There was no information on possible contact to tourists available.

**Table 1:** Demographic characteristics of the study population for the full time period.

	**Quarantined persons *n* (%)**	**PCR confirmed cases *n* (%)**
**Total**	7,440	611
**Gender**		
Female	3,531 (50.7)	290 (47.5)
Male	3,429 (49.3)	319 (52.3)
Diverse	2 (0.03)	1 (0.2)
Missing	478 (6.4)	1 (0.2)
**Age**		
Mean (SD)	32.6 (20.6) years	38.2 (21.3)
Median (range)	30 (0–100) years	36 (1–93)
Missing	201 (2.7)	4 (0.7)
**Location**		
Mainland	6,337 (85.2)	518 (84.8)
Islands	1,103 (14.8)	93 (15.2)
**Measures**		
Quarantine	6,634 (89.2)	607 (99.3)
Isolation	806 (10.8)	4 (0.7)

Overall, 1–184 PCR confirmed cases (median: 3) were detected in 74 parishes (55.6%). The highest number of cases was reported for *Husum* (184), followed by *Gemeinde Sylt* (49), *Niebüll*, and *Viöl* (31 each). For clearer representation, parishes were summarized into their respective administrative areas. The highest proportion of quarantined persons was reported for *Husum* (26.2%), followed by *Südtondern* (17.6%), *Nordsee-Treene* (13.5%), and *Mittleres Nordfriesland* (13.2%). The lowest proportion of quarantined persons was reported for *Pellworm* (0.4%). The most PCR-confirmed cases were reported for *Husum* (*n* = 184, 30.1%). Only six confirmed cases were reported for *Pellworm* (1.0%; Table 2). Reports of confirmed cases in each administrative area was low during summer and a rise in case numbers occurred in August and September 2020 (Supplementary Figure 1).

**Table 2:** Geographic distribution of SARS-CoV-2 cases and quarantined persons in North Frisian administrative areas.

	**Quarantined persons *n* (%)**	**PCR confirmed cases *n* (%)**
**Total**	**7,440**	**611**
*Eiderstedt*	487 (6.5)	37 (6.1)
*Föhr-Amrum*	274 (3.7)	23 (3.8)
*Husum*	1,950 (26.2)	184 (30.1)
*Mittleres Nordfriesland*	984 (13.2)	77 (12.6)
*Nordsee-Treene*	1,003 (13.5)	65 (10.6)
*Pellworm*	33 (0.4)	6 (1.0)
*Südtondern*	1,312 (17.6)	107 (17.5)
*Sylt*	796 (10.7)	64 (10.5)
*Viöl*	601 (8.1)	48 (7.9)

### SARS-CoV-2 infections among tourists and returning travelers

**Figure 3:**
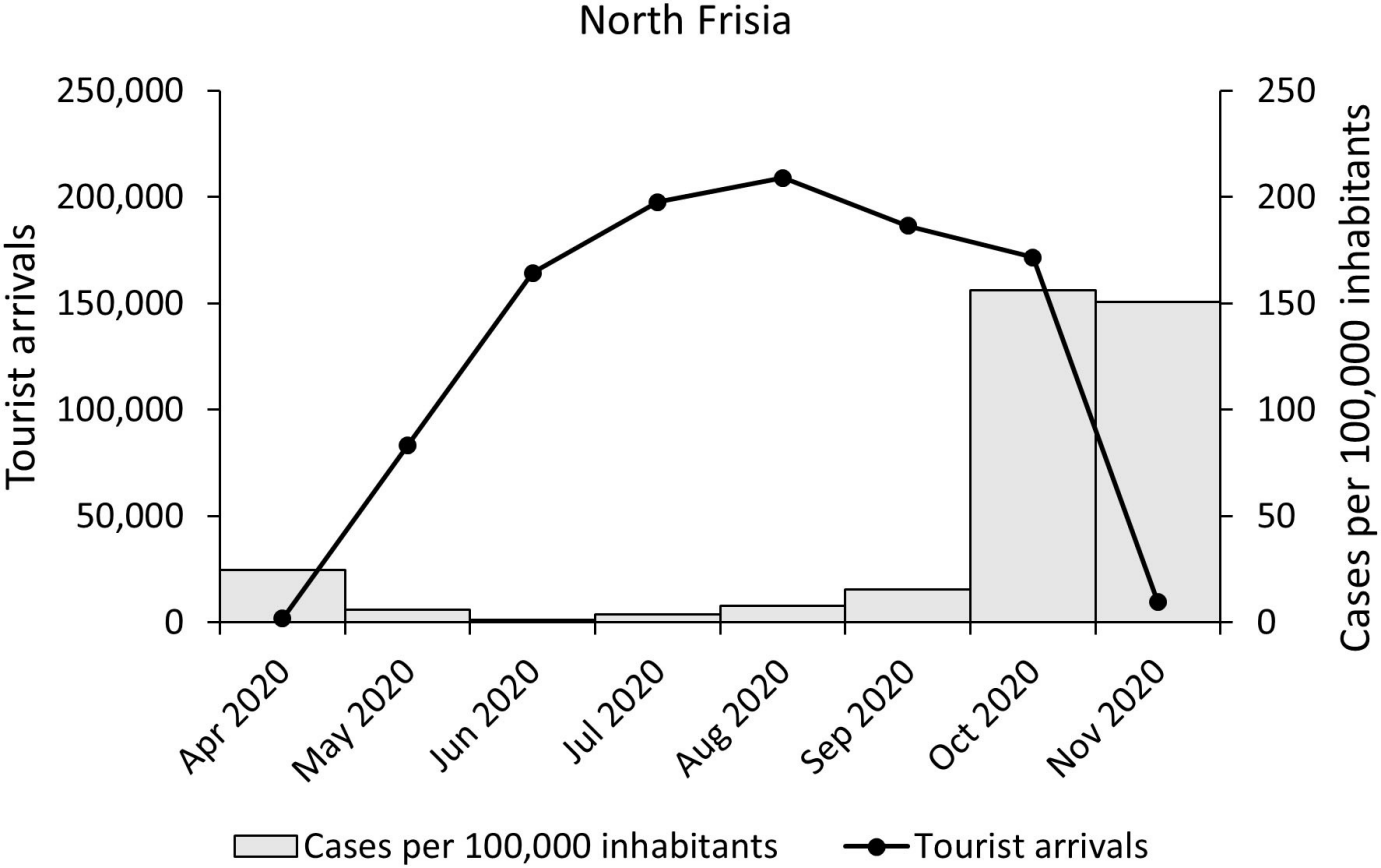
Tourist arrivals and SARS-CoV-2 cases. Shown are PCR-confirmed cases of SARS-CoV-2 infections (gray colums) and tourist arrivals (black line) per month in North Frisia.

In 2020, at least 1,264,139 tourists arrived in North Frisian hotels, not including camping and summer residences. Hotels were shut down in mid-March and April 2020 and re-opened in May 2020 until they were closed again in November 2020. Tourist arrivals were highest between June and August. SARS-CoV-2 cases began to rise markedly in October (Figure 3). The pattern was similar for each administrational region except for *Viöl*, where no tourists were reported (Supplementary Figure 1). Overall, hotels in 46 North Frisian parishes offered beds in 2020. The number of hotels with tourists ranged from 3 (e.g., *Bredstedt*) to 251 hotels in *Sylt*. The largest number of arrivals were recorded in *Sylt* (373,445 arrivals, 29.5%), followed by *Sankt Peter-Ording* (234,718 arrivals, 18.6%), *Wenningstedt-Braderup/Sylt* (104,161 arrivals, 8.2%), *Wyk/Föhr* (96,389 arrivals, 7.6%), and *Husum* (56,055 arrivals, 4.4%). Overall, 65% of the tourists visited the islands of North Frisia (Sylt, Föhr-Amrum, Pellworm). Tourists stayed in North Frisian hotels on average for 6.4 days (SD: 2.3 days). The majority of tourists were German (93.3%) followed by tourists from other European countries (6.4%).

In the dataset a total of 34 persons were labeled as tourists from other districts (0.5%), out of whom none tested positive for SARS-CoV-2. One thousand three hundred sixty-two persons were labeled as returning travelers (18.3%). Out of these, 13 persons were PCR-confirmed cases (1.0%). No tourism-related outbreaks were reported.

### Transmissions

Original information on where persons had contact to SARS-CoV-2 cases was sparse (*n* = 612, 8.2%). Assumed transmission among quarantined persons was reported for school/kindergarten (31.8%), followed by gastronomy (19.8%) and medical settings (14.1%). Among cases, mostly transmission in school/kindergarten or household transmissions were reported (52.9% and 38.2%).

The information on a possible setting for infection was given for 83.2% of observations (*n* = 6,188), where the largest group with a specific context worked or was present in an educational setting (46.4%), and mostly children/students were affected (39.8%) with 181 PCR-confirmed cases. *Other work settings* comprised the second largest group (30.6%; Table 3).

**Table 3:** Possible settings of transmission.

**Setting**	**Quarantined persons *n* (% of 6,188)**	**Cases *n* (% of 513)**
**Tourism and gastronomy**	**320 (5.2)**	**42 (8.2)**
Hotel	96 (1.6)	11 (2.1)
Other tourism	11 (0.2)	2 (0.4)
Gastronomy	213 (3.4)	29 (5.7)
**Medicine**	**416 (6.7)**	**38 (7.4)**
Staff	414 (6.7)	38 (7.4)
Patients	2 (0.03)	0 (0)
**Nursing homes**	**274 (4.4)**	**18 (3.5)**
Staff	221 (3.6)	11 (2.1)
Residents	53 (0.9)	7 (1.4)
**Education**	**2,870 (46.4)**	**181 (35.3)**
Staff	408 (6.6)	27 (5.3)
Children and students	2,462 (39.8)	154 (30.0)
**No work**	**414 (6.7)**	**56 (10.9)**
Retired	302 (4.9)	44 (8.6)
Unemployed	60 (1.0)	6 (1.2)
Other groups^*^	52 (0.8)	6 (1.2)
**Other work settings**	**1,894 (30.6)**	**178 (35.0)**
**Missing data**	**1,252 (16.8)**	**98 (16.0)**

* Other groups include persons in parental leave, sick leave, holidays, as well as infants.

**Figure 4:**
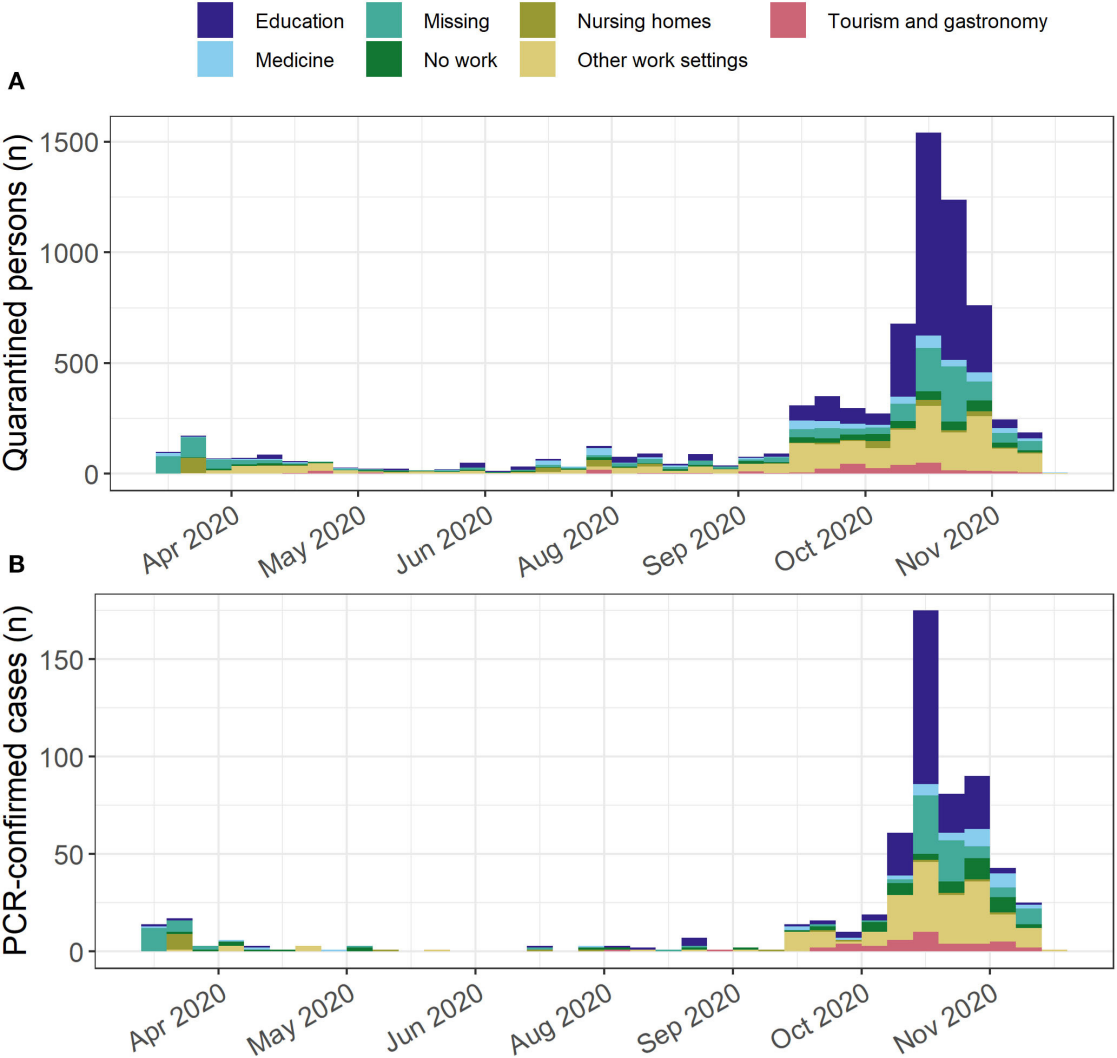
Epidemic curve stratified by setting. SARS-CoV-2 epidemic curve for North Frisia stratified by setting for **(A)** all quarantined persons and **(B)** PCR-confirmed cases.

The proportion of quarantined persons working in the *Tourism and gastronomy* setting was small (*n* = 320, 5.2%). Of these, 42 persons were confirmed cases (13.1%). The number of quarantined persons and PCR-confirmed cases working in the *Tourism and gastronomy* setting was most pronounced around October 2020 (Table 3, Figure 4).

For 2,990 adult persons the occupation could be classified according to the *ISCO-08* system (40.2%). Professionals and therein teaching professionals comprised the largest group (23.2 and 14.2%, respectively). Associate professionals such as nurses and service and sales worker followed with 22.6 and 16.7%, respectively. Interestingly, craft and related trades were a relatively large group (16.2%), and therein people working in food processing, e.g., butchers and workers in meat-processing, were common (5.5%). Among PCR-confirmed cases (*n* = 271), craft and related trades worker comprised the largest group (21.4%), followed by professionals (19.9%), service and sales worker (19.2%), and technician/associate professionals (14.8%; Table 4).

**Table 4:** SARS-CoV-2 cases and quarantined persons stratified by occupation.

	**Quarantined persons**	**PCR confirmed cases**
	***n* (% of 2,990)**	***n* (% of 271)**
**0 Armed forces occupations**	**87 (2.9)**	**5 (1.8)**
**1 Managers**	**84 (2.8)**	**10 (3.7)**
14 Hospitality managers	39 (1.3)	6 (2.2)
1,411 Hotel managers	7 (0.2)	0 (0)
1,412 Restaurant managers	32 (1.1)	6 (2.2)
**2 Professionals**	**694 (23.2)**	**54 (19.9)**
22 Health professionals	131 (4.4)	18 (6.6)
23 Teaching professionals	425 (14.2)	23 (8.5)
**3 Technician/associate professionals**	**677 (22.6)**	**40 (14.8)**
32 Health associate professionals	311 (10.4)	19 (7.0)
**4 Clerical support workers**	**152 (5.1)**	**17 (6.3)**
422 Client information workers^*^	13 (0.4)	2 (0.7)
**5 Services and sales workers**	**500 (16.7)**	**52 (19.2)**
51 Personal services workers	278 (9.3)	28 (10.3)
513 Waiters and bartenders	91 (3.0)	9 (3.3)
**6 Skilled agricultural workers etc**.	**48 (1.6)**	**4 (1.5)**
**7 Craft and related trade workers**	**484 (16.2)**	**58 (21.4)**
71 Building and related trades	173 (5.8)	22 (8.1)
75 Food processing and related trades	164 (5.5)	23 (8.5)
**8 Plant and machine operators**	**68 (2.3)**	**9 (3.3)**
**9 Elementary occupations**	**196 (6.6)**	**22 (8.1)**
9,112 Cleaners and helpers in offices, hotels and other establishments	91 (3.0)	9 (3.3)

* Including hotel receptionists and general receptionists. The bold letters indicated to highlight the main groups.

Occupational groups with relation to hotels or gastronomy had only small proportions. For example, waiters and bartenders comprised 3.0% of quarantined persons and 3.3% of PCR-confirmed cases, and receptionist comprised only 0.4% of quarantined persons and 0.7% of PCR-confirmed cases (Table 4).

## Discussion

### General findings

The contribution of tourism to the SARS-CoV-2 infection dynamics in the district of North Frisia in Germany during the tourism season 2020 was studied based on routine data from the local health authority. This district is of interest to research, as it has a low population density and a high number of visiting tourists.

The overall low incidences during summer and the peak in October/November 2020 reflect the country-wide infection dynamics [Figures 1, 2 (4)]. The time course of the case numbers is presumably affected by countermeasures and a seasonal effect. A simulation study performed with data from May 2020 to November 2020 found that around 50% of COVID-19 cases are related to seasonality, where both infectivity and mortality are higher in colder climates (22). A more recent study which included 29 European countries found a seasonal effect comparable to non-pharmaceutical interventions (23).

### Tourists among cases

Even though touristic activities played a prominent role in the worldwide spread of the SARS-CoV-2 pandemic, there were no tourism-related outbreaks reported in North Frisia in the study period. Only a small proportion of quarantined persons and no PCR-confirmed case were labeled as tourists in the dataset. However, it is to note that rapid tests were not available in summer 2020 and that tourists just as other individuals were not systematically tested. As well, tourists stayed on average for around 6 days in North Frisia, which roughly overlaps with the incubation time of the virus and infected persons might have noticed symptoms only after arriving back at home. This implies that possible cases were not or not always reported to the health authorities in North Frisia.

### Contribution of tourism to infection dynamics

Incidences in North Frisia were mostly lower compared to the average incidences in Germany. As the patterns of tourist arrivals in comparison to the case numbers were similar in districts with low tourism, e.g., *Mittleres Nordfriesland*, and in districts with high tourism, e.g., *Sylt* (Supplementary Figure 1), there is no clear indication of a substantial contribution of tourism to the SARS-CoV-2 infection dynamics. Even though the majority of tourists visited the islands of North Frisia, case numbers on the islands were not over-represented, i.e., the majority of the cases were inhabitants of the mainland (85%). However, the rise of incidences in September and October 2020 occurred while tourist arrivals were still high and comparable to the respective period in 2019 (Supplementary Figure 2). Countrywide, an increase of incidences due to traveling during summer school holidays has been shown by Plümper and Neumayer (24). The authors found an average increase of around 45% in the district incidences at the end of the holidays and 2 weeks later. Therefore, a contribution of traveling to rising case numbers cannot be excluded. Still, it is notable that the case numbers were low for most of summer when tourist arrivals peaked. This may be due to several reasons. First, incidences were relatively low in summer in most districts. Second, after the shut down in March/April 2020 NPI such as mandatory mask wearing and social distancing were in place and mass events were prohibited. As well, cautious behavior might have played a role. Third, virus variants with higher infectivity were not present in the tourism period 2020. However, it is to note that systematic screening for virus variants only began in early 2021.

Other factors such as the tourist clientele or the mode of transport (air plane vs. car) might have played a role. Studies analyzing SARS-CoV-2 outbreaks or transmissions in a touristic setting were mostly done for international air travel and cruise ships. For example, in a case study the authors reconstructed a SARS-CoV-2 transmission from a hotel manager in Israel to seven members of a tourist group (in total 24 persons) who later presumably infected other passengers on a flight to Germany (12). More than 800 COVID-19 cases and at least 10 deaths occurred on three cruise ship voyages and both passengers and crew were affected. Additional transmissions spread further to other ships by crew members (13).

### Site of infections

The proportion of quarantined persons and PCR-confirmed cases related to a touristic setting was relatively small overall (~5 and 8%) indicating a small contribution of touristic activity to the infections. The main settings in which infections possibly occurred were settings without tourist contact (Table 3, Figure 4). The site of infections could not be clearly identified from the reports in the dataset due to a large amount of missing data. However, the data indicate that household transmissions might be relevant as has been shown in other studies (25).

Relevant settings in which outbreaks and transmissions occurred were educational settings, nursing homes, hospitals and a meat-processing factory, i.e., indoor settings in which many persons gather (Tables 3, 4). Overall, schools and kindergartens in North Frisia were largely affected by outbreaks.

### Strengths and limitations

The data for this study contained more information than usually publicly available, e.g., information on occupation or where individuals had contact with others, such that possible transmission routes could be inferred. However, as only data from one local authority was available, it is possible that a number of cases among tourists might have occurred without being noticed, as it is unclear, if other health authorities reported back all cases of persons who visited North Frisia prior to a positive test result. Additionally, in 2020 tourists were not yet systematically rapid tested for SARS-CoV-2 infection and a negative test result at arrival was not mandatory. Overall, the amount of cases might be higher than reported and varied between first and second wave (26). Thus, case numbers throughout the time course are not comparable. In this study, the number of tourists visiting North Frisia in 2020 might also be underestimated, as the tourism data only included overnight stays in hotels with at least 10 beds. As well, due to travel restrictions fewer international tourists arrived in North Frisia compared to other seasons and mostly German tourists visited the district.

The “setting of transmission” was derived from the information given on occupation and place of work and is not a standardized procedure, but a crude method in order to identify settings in which infections might occur. The choice of settings does not reflect the chance for transmission. For example, a janitor working in a hospital might have a lower risk for infection than a nurse. However, as the overall size of the occupational groups is unknown, it is not possible to calculate risks. Thus, we cannot state, if persons who work in a specific sector were at increased risk compared to other sectors.

There was no information on where the deceased persons got infected. A potential contact with tourists cannot be excluded. Therefore, a statement on safety cannot be drawn.

## Conclusion

During the tourist season 2020, it seemed that tourism did not contribute substantially to SARS-CoV-2 infection dynamics in North Frisia. Local incidence figures were presumably not increased by the arrival of large numbers of tourists and tourism-related outbreaks were not reported. However, during the summer holidays season incidences were overall low in the whole of Germany. Additionally, individual NPI, such as mandatory mask wearing and social distancing, were in place, although large scale community testing was not. Overall, causal relationships cannot be drawn from this descriptive study.

In summary, while in 2020 it was apparently safe to spend holidays in North Frisia for tourists, neither did they pose a risk to the resident population. It would be of interest to compare the results with the developments in the tourist season 2021, in which the more transmissible virus strain, the so-called *Delta* variant (27), is spreading, vaccines and rapid antigen tests are widely available, and tourism-specific protective measures, such as negative test results or vaccine certificates on arrival are mandatory.

## Data availability statement

The data in this manuscript is routine data from the Health Authority of the district North Frisia. This data contains more details than is publicly available in the routine data. Requests to access these datasets should be directed at: timogreiner@googlemail.com.

## Ethics statement

The project was approved by the Ethics Committee of the Charité Universitätsmedizin Berlin (Nr.118 EA2/093/21).

## Author contributions

TG analyzed the data. AA and PT supervised the project. PT designed the project. TG, AA, and PT interpreted the data and wrote the manuscript. All authors contributed to the article and approved the submitted version.

## Conflict of interest

The authors declare that the research was conducted in the absence of any commercial or financial relationships that could be construed as a potential conflict of interest.

## Publisher's note

All claims expressed in this article are solely those of the authors and do not necessarily represent those of their affiliated organizations, or those of the publisher, the editors and the reviewers. Any product that may be evaluated in this article, or claim that may be made by its manufacturer, is not guaranteed or endorsed by the publisher.
