# Supplementary material for: SARS-CoV-2 infection dynamics in the tourism season 2020 in North Frisia, Germany
Source: Front Epidemiol. 2022 Dec 1;2:1029807. doi: 10.3389/fepid.2022.1029807 (PMC10910935; doi:10.3389/fepid.2022.1029807)
Supplement: Supplementary file 1 [file Data_Sheet_1.PDF]

Supplemental figure 1 A-H. Tourist arrivals and cases per administrational area. Tourist arrivals (black line) and PCR confirmed cases per 100.000 inhabitants (grey columns) per month for each administrational area for the whole time period. There were no reported tourist arrivals in *Viöl*.

A

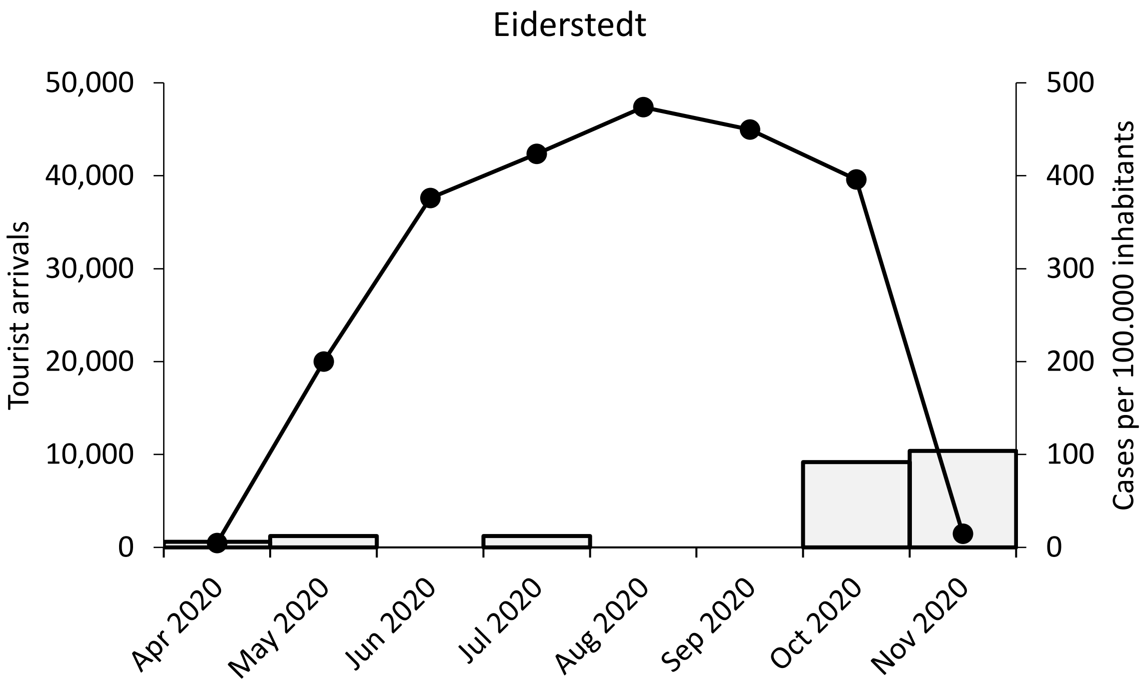

B

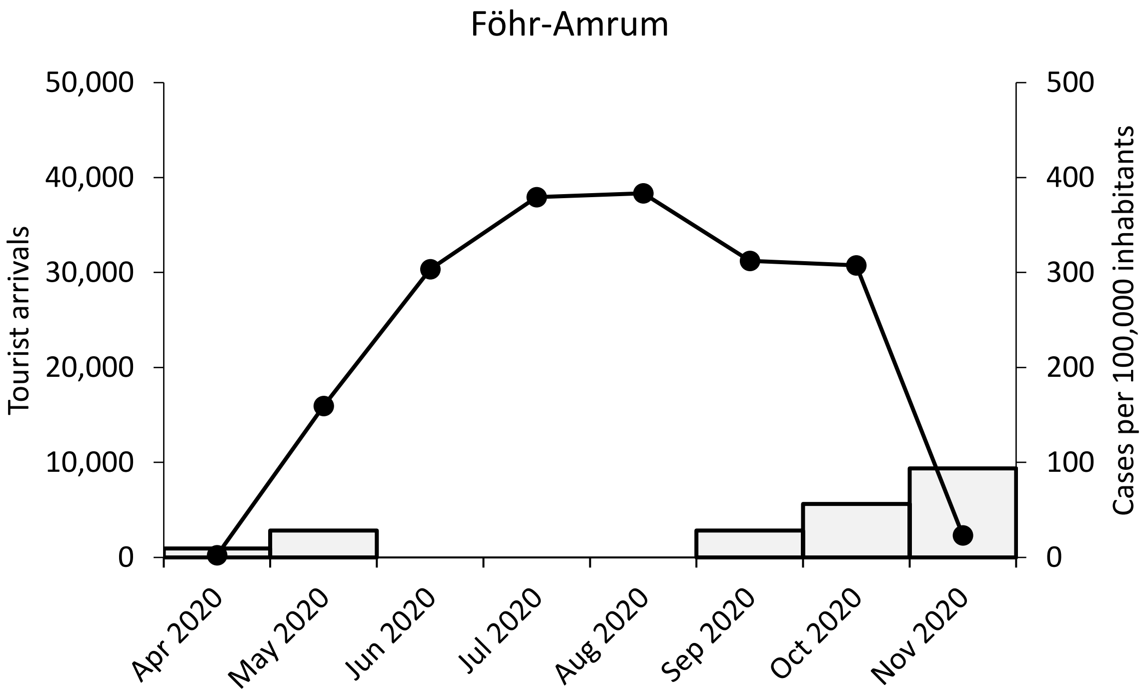

C

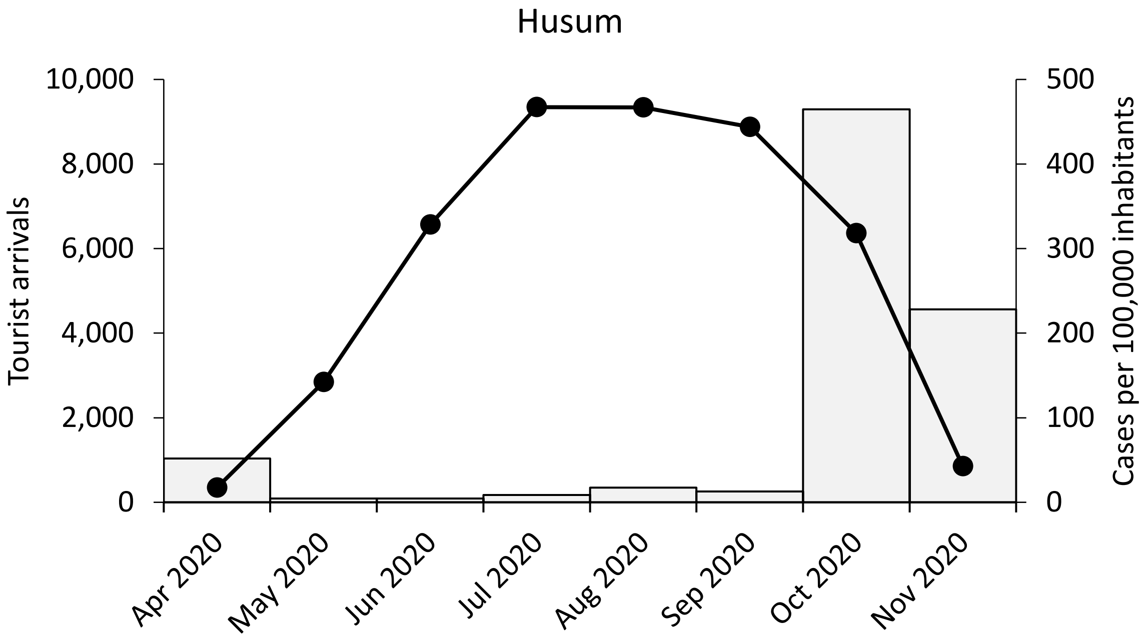
Husum
Tourist arrivals
Cases per 100,000 inhabitants
10,000
8,000
6,000
4,000
2,000
0
500
400
300
200
100
0
Apr 2020
May 2020
Jun 2020
Jul 2020
Aug 2020
Sep 2020
Oct 2020
Nov 2020

D

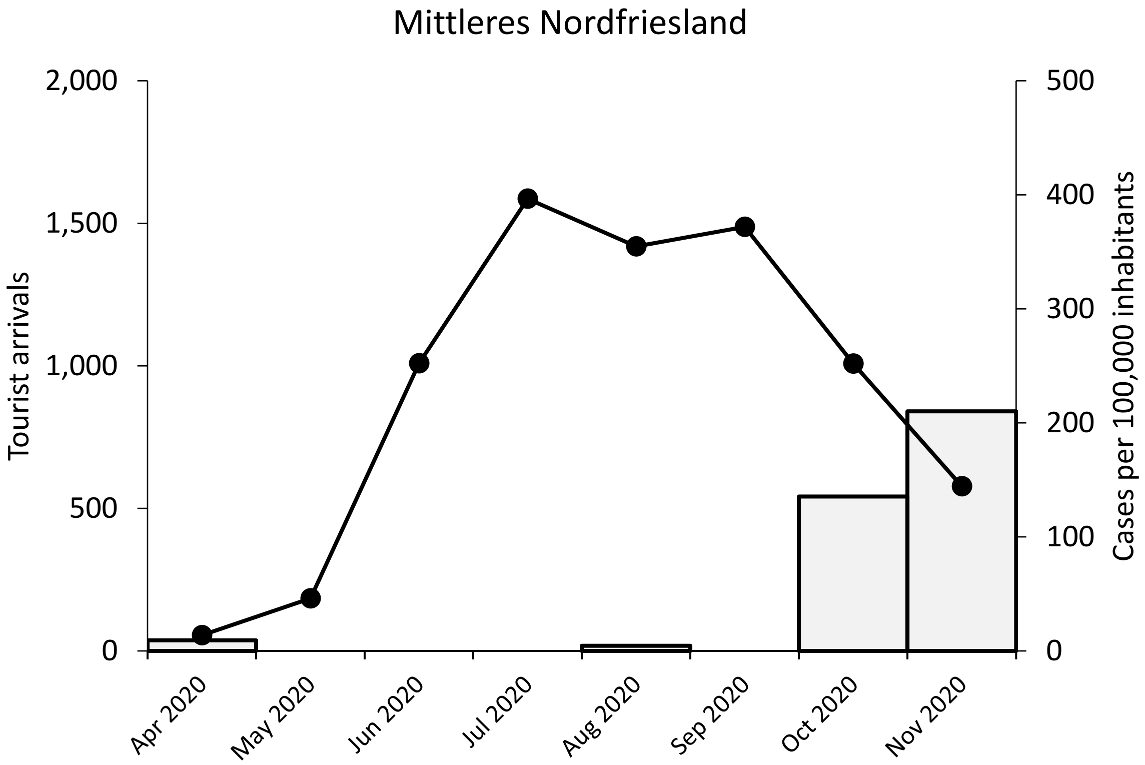
Mittleres Nordfriesland
Tourist arrivals
Cases per 100,000 inhabitants
2,000
1,500
1,000
500
0
500
400
300
200
100
0
Apr 2020
May 2020
Jun 2020
Jul 2020
Aug 2020
Sep 2020
Oct 2020
Nov 2020

E

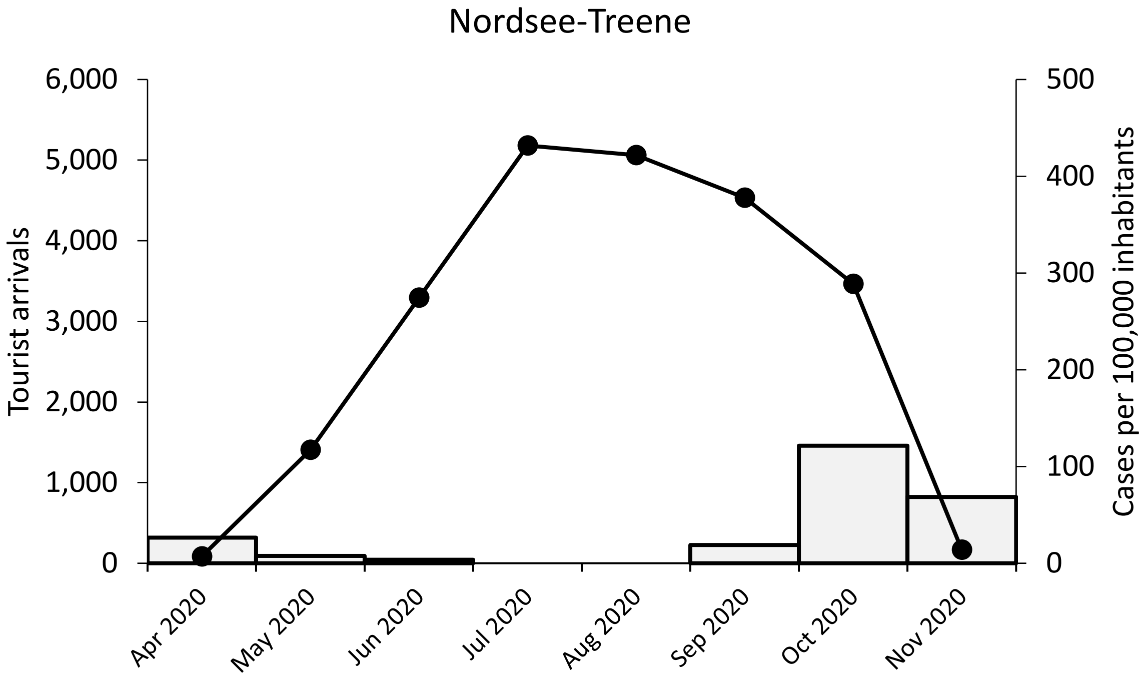
Nordsee-Treene
6,000
5,000
4,000
3,000
2,000
1,000
0
Tourist arrivals
500
400
300
200
100
0
Cases per 100,000 inhabitants
Apr 2020
May 2020
Jun 2020
Jul 2020
Aug 2020
Sep 2020
Oct 2020
Nov 2020

F

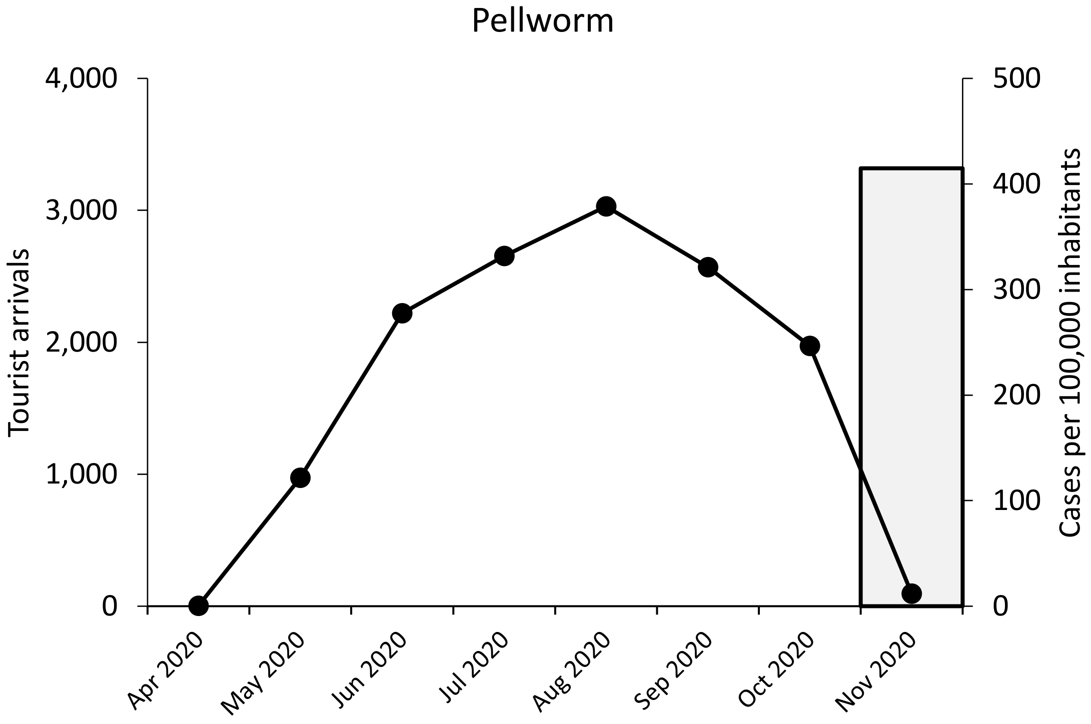
Pellworm
4,000
3,000
2,000
1,000
0
Tourist arrivals
500
400
300
200
100
0
Cases per 100,000 inhabitants
Apr 2020
May 2020
Jun 2020
Jul 2020
Aug 2020
Sep 2020
Oct 2020
Nov 2020

G

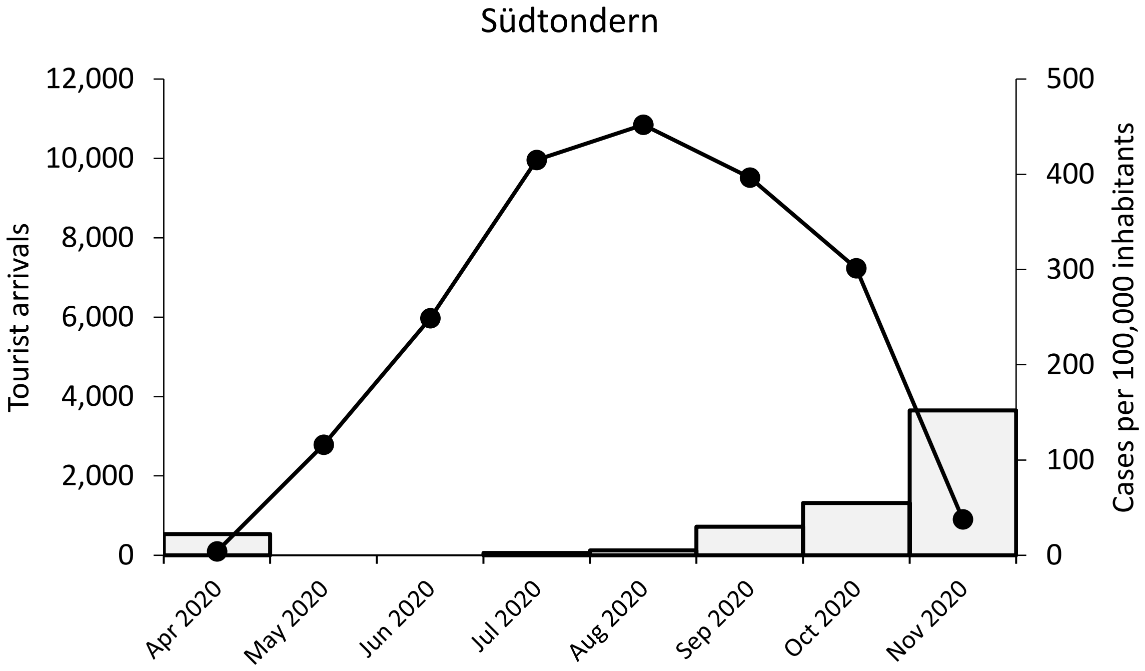
Südtondern
Tourist arrivals
Cases per 100,000 inhabitants
12,000
10,000
8,000
6,000
4,000
2,000
0
500
400
300
200
100
0
Apr 2020
May 2020
Jun 2020
Jul 2020
Aug 2020
Sep 2020
Oct 2020
Nov 2020

H

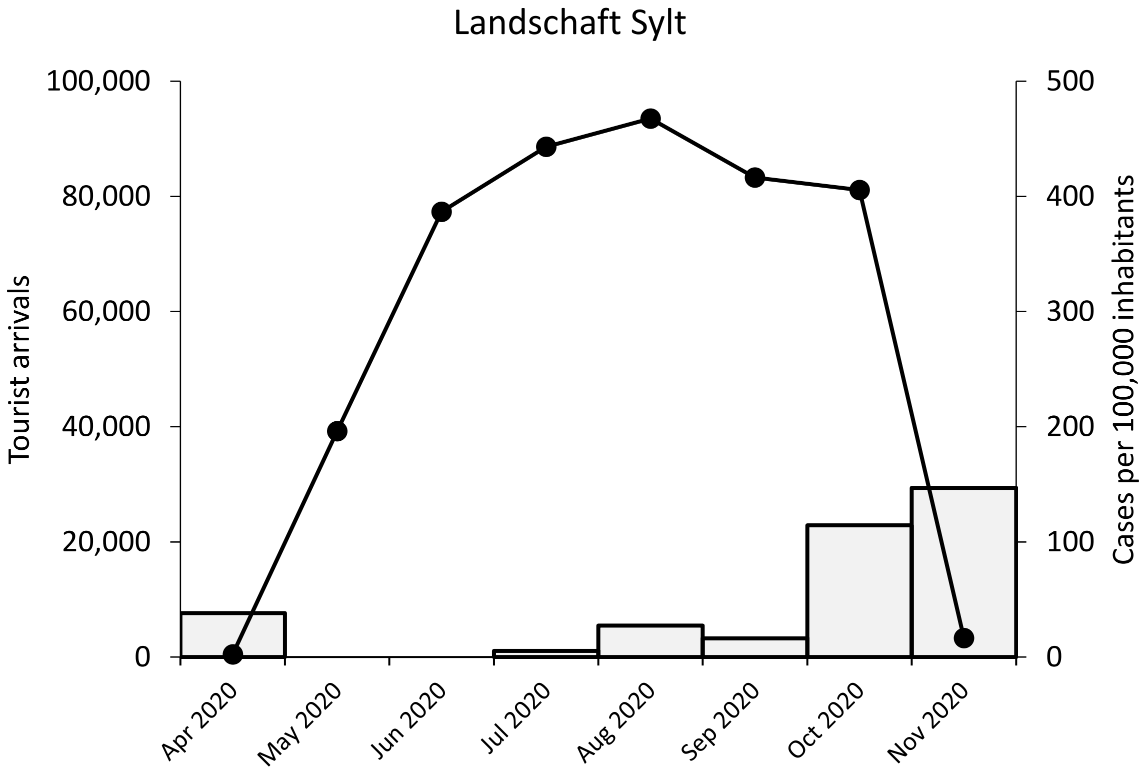
Landschaft Sylt
Tourist arrivals
Cases per 100,000 inhabitants
100,000
80,000
60,000
40,000
20,000
0
500
400
300
200
100
0
Apr 2020
May 2020
Jun 2020
Jul 2020
Aug 2020
Sep 2020
Oct 2020
Nov 2020

I

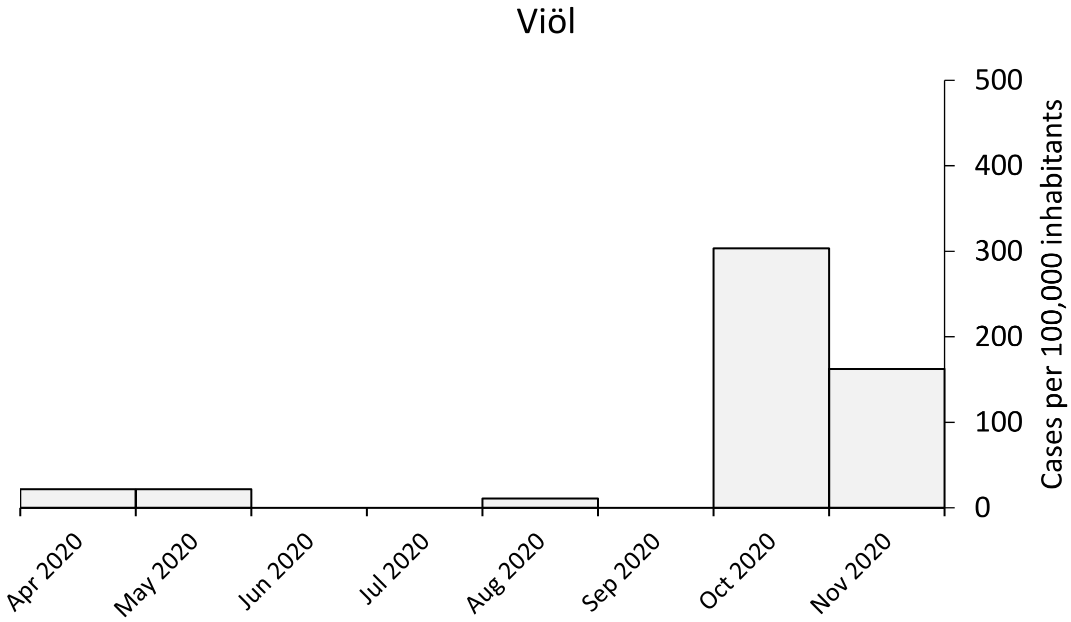

Viöl
500
400
300
200
100
0
Cases per 100,000 inhabitants
Apr 2020
May 2020
Jun 2020
Jul 2020
Aug 2020
Sep 2020
Oct 2020
Nov 2020

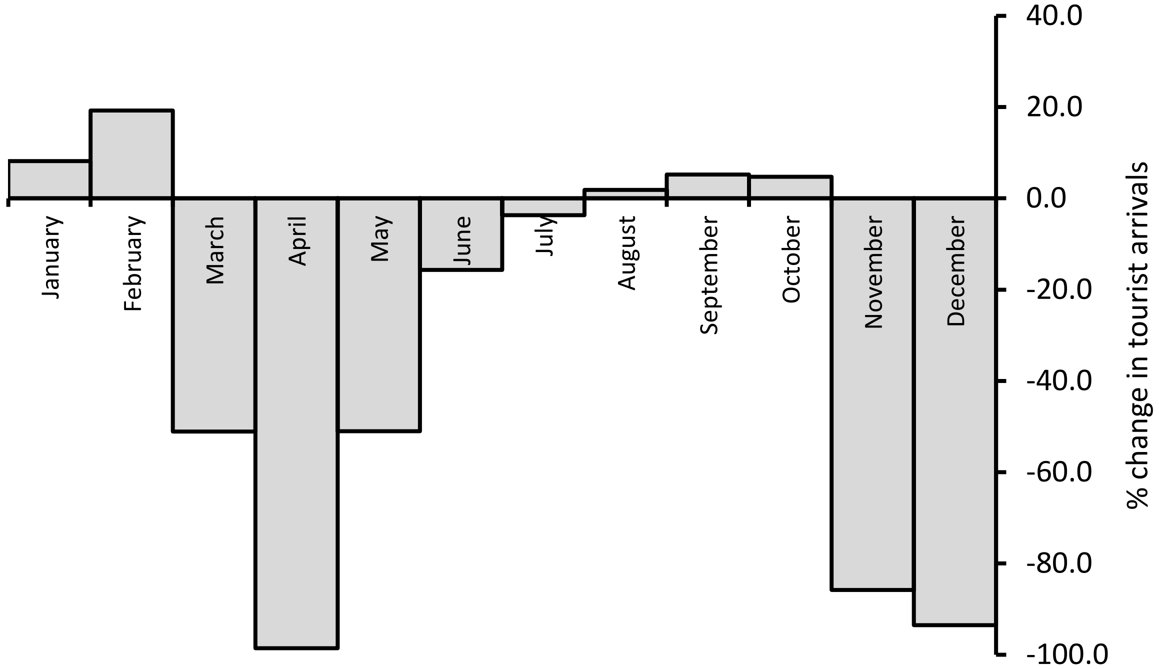

Supplemental figure 2. Tourist arrival changes. Percent change in tourist arrivals in 2020 vs. 2019
